# Hypoechogenicity of brainstem raphe in long-COVID syndrome–less common but independently associated with depressive symptoms: a cross-sectional study

**DOI:** 10.1007/s00415-022-11154-3

**Published:** 2022-05-12

**Authors:** Daniel Richter, Hannah Schulze, Jeyanthan Charles James, Nadine Siems, Nadine Trampe, Ralf Gold, Christos Krogias, Simon Faissner

**Affiliations:** 1 Department of Neurology, St. Josef-Hospital, Ruhr-University Bochum, Bochum, Germany; 2 Medical Faculty, Ruhr-University, Bochum, Germany

## Abstract

**Objective:**

Long coronavirus disease (Long-COVID) syndrome is a hitherto poorly understood phenomenon with a broad spectrum of symptoms, including depression and anxiety. Depressive symptoms have been associated with brainstem raphe (BR) alterations in transcranial sonography (TCS) that might reflect dysfunction of the serotonergic system. The primary aim was to investigate the connection of BR alterations with depressive and anxiety symptoms in patients with Long-COVID syndrome.

**Methods:**

In a cross-sectional study design, we included outpatients fulfilling the criteria of Long-COVID syndrome. All patients were examined by TCS in the axial plane with focus on BR signal alterations. The Hospital Anxiety and Depression Scale (HADS) was used to test for symptoms of anxiety and depression.

**Results:**

We included *n* = 70 patients with Long-COVID syndrome, of which 28.6% (*n* = 20) exhibited a reduced echogenicity of BR in the TCS examination. Patients with hypoechogenic BR had higher subscores for anxiety and depression compared to normoechogenic patients (HADS depression: median 8 versus 5.5, *p* = 0.006; HADS anxiety: median 9 versus 6.5, *p* = 0.006). After adjustment for reasonable confounders, only the odds ratio (OR) for relevant depressive symptoms was higher among Long-COVID patients with hypoechogenic raphe (adjusted OR 3.884, 95% CI 1.244–12.123).

**Discussion:**

Hypoechogenic BR alterations are independently associated with depressive symptoms in Long-COVID patients but are not highly frequent. Future studies should investigate whether the hypoechogenicity of the BR is a direct consequence or whether it reflects a priori a higher susceptibility to depressive symptoms after COVID-19, thus enabling to identify COVID-19 patients at higher risk of developing Long-COVID depressive symptoms.

**Supplementary Information:**

The online version contains supplementary material available at 10.1007/s00415-022-11154-3.

## Introduction

Being initially reported as a pure respiratory infection, it became quickly apparent that Coronavirus disease 2019 (COVID-19) is a multi-organ disease, giving rise to cardiovascular, renal, gastrointestinal, hepatic, hematological, metabolic, and neurological disorders [1–3]. After COVID-19, many patients of varying ages reported a broad spectrum of ongoing or newly developed symptoms, which led to the concept of Long-COVID syndrome. As a poorly understood phenomenon, there is a tremendous scientific effort to improve our knowledge of pathophysiology and identify risk factors for Long-COVID syndrome.

The broad spectrum of Long-COVID includes pulmonary symptoms such as cough and shortness of breath but also more generalized symptoms including fatigue, physical limitations, depression, and anxiety. Indeed, depression and anxiety are major consequences of COVID-19 which are present in 23% of patients six months after acute infection [4]. In other diseases, depressive symptoms have been associated with brainstem raphe (BR) alteration detected by transcranial sonography (TCS) [5–8]. A reduced echogenic signal of the BR in TCS is thought to visualize a dysfunction in the serotonergic system that might be a substrate of depressive symptoms [9]. In COVID-19, it has been postulated that modifications of the dopamine and serotonin synthetic pathways contribute to COVID-19 pathophysiology, as gene co-expression, co-regulation, and function are linked between Angiotensin I Converting Enzyme 2 (ACE2) and Dopa Decarboxylase (DDC) [10, 11].

As we hypothesized that BR alteration might be more frequent in patients with Long-COVID, the primary aim of this study was to investigate the frequency of BR alterations in TCS and its connection to depressive and anxiety symptoms in patients with Long-COVID syndrome.

## Methods

### Study design and participants

In a cross-sectional study design, we prospectively included patients in the period between January to October 2021 who presented to our outpatient clinic fulfilling the criteria for Long-COVID syndrome [12], which was defined as:

- PCR-confirmed COVID-19 disease at least 12 weeks ago.
- Symptoms persisting from COVID-19 ***or*** newly occurred symptoms after the acute phase, considered as sequelae of COVID-19.

Participants with an insufficient transtemporal bone window were excluded. All patients underwent clinical neurological assessment and TCS examination on the same day. Demographic and clinical data were obtained, including the onset of COVID-19 and comorbidities. Initial COVID-19 severity was classified according to the World Health Organization (WHO) clinical progression scale [13; table S1].

### Outcome parameters

The presence of depressive and anxious symptoms was investigated by the German version of the self-assessment Hospital Anxiety and Depression Scale (HADS) [14].

**Fig. 1:**
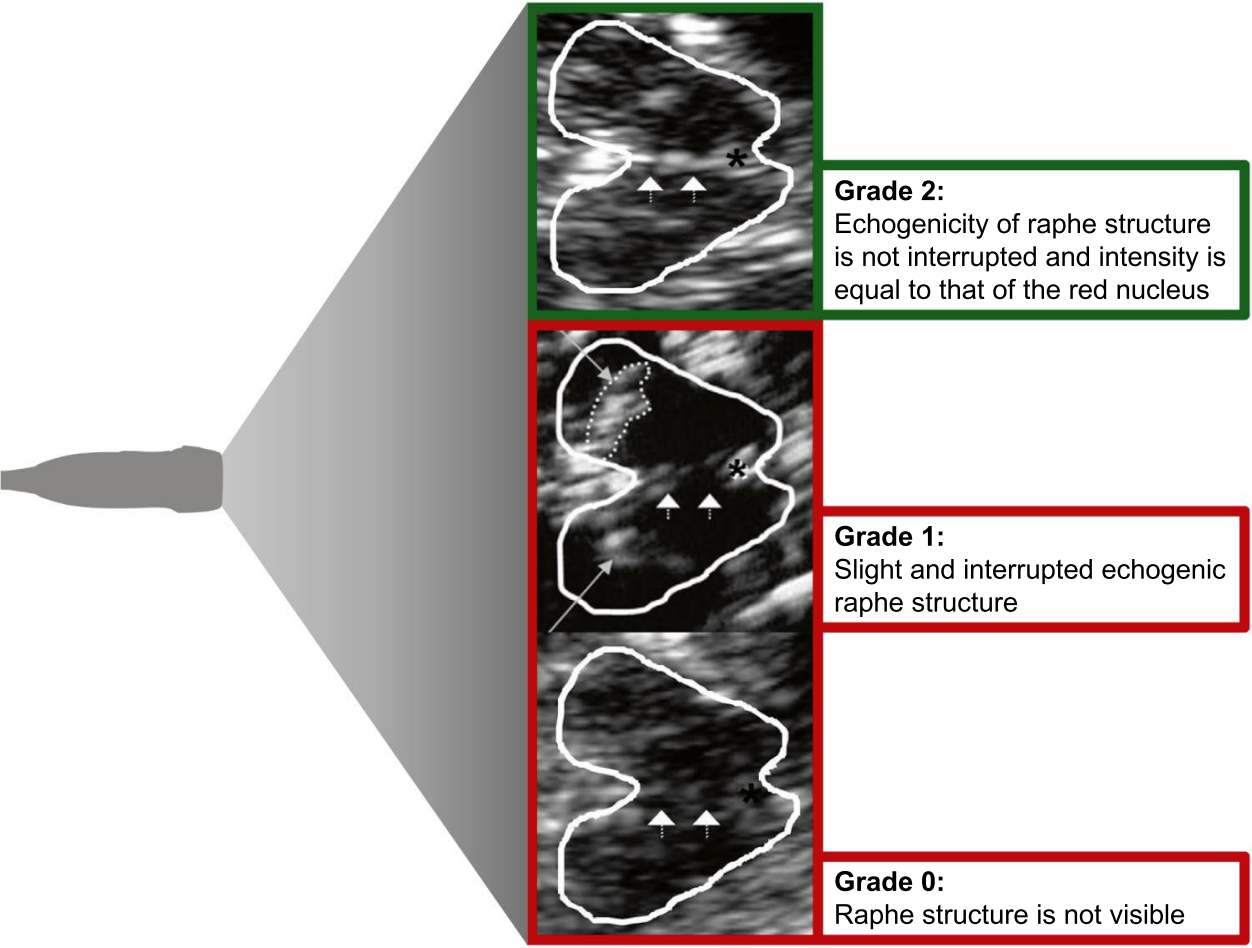
Raphe grading in TCS. Enlarged image of the brainstem raphe in three different patients using the mesencephalic axial examination plane. The butterfly-shaped midbrain is outlined for better visualization. The asterisk indicates the aqueduct. Arrowheads indicate the brain stem raphe. Long arrows indicate the hyperechogenic enlarged area of Substantia nigra. Raphe grading: (Grade 2) Normal echogenicity, normal finding, outlined in green; (Grade 1) Echogenic line of the raphe is interrupted, pathological finding, outlined in red; (Grade 0) Raphe structure not visible, pathological finding, outlined in red

The transcranial sonographic examination was performed by two experienced investigators (DR 7 years and CK more than 20 years of TCS experience). The investigators were blinded to clinical scores and the patient's history. We used a phased array ultrasound system equipped with a 2.5-MHz transducer (Aplio XG Ultrasound System, Toshiba Medicals, Tochigi, Japan). A penetration depth of 150 mm and a dynamic range of 45–50 dB were chosen. Image brightness and time gain compensations were adapted as needed for each examination. The examination protocol was based on previously published recommendations for TCS [15]. Using the transtemporal approach, the midbrain and diencephalic examination planes were visualized in the axial section. The grade of BR echogenicity was assessed semi-quantitatively on a three-point scale (0 = raphe structure not visible, 1 = slight and interrupted echogenic raphe structure, 2 = normal echogenicity) and dichotomized for further analysis into normoechogenic (echogenicity of raphe structure is not interrupted and intensity is equal to that of the red nucleus) and hypoechogenic (interrupted or absent raphe structure, Fig. 1) [9].

The sonographic findings were stored to perform a second evaluation and classification of the results by the second investigator. In the case of discrepant ratings, a consensus was accomplished subsequently.

### Statistical analysis

Rates are given for categorical variables and median and interquartile range for continuous variables. We compared demographics, clinical characteristics, and outcomes of hypoechogenic versus normoechogenic patients with univariate analysis using appropriate nonparametric tests (Mann–Whitney-*U* test, Chi-squared test). Furthermore, we applied a logistic regression model to calculate odds ratios (OR) and the corresponding 95% confidence intervals (CI) for the outcome of depression and anxiety with BR hypoechogenicity as the predictor. A value of ≥ 8 points in the subscales of HADS was used as cut-off to indicate relevant depressive or anxiety symptoms, respectively. OR are given unadjusted and after adjustment for COVID-19 severity and comorbid psychiatric or psychosomatic disease, which is known to be associated with BR hypoechogenicity [9], as well as for significant demographic differences. Interrater agreement was analyzed by Cohen's kappa statistics.

Statistical analysis was performed with SPSS 27.0 for Mac. *p* < 0.05 was defined as the level of statistical significance.

## Results

### Study population

**Fig. 2:**
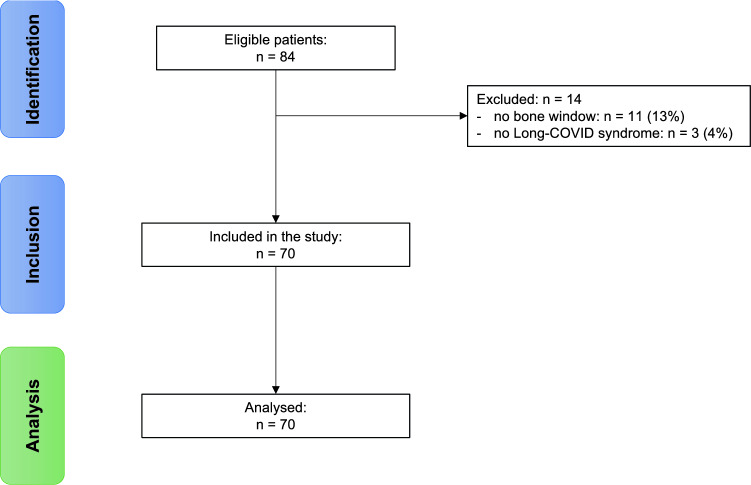
Study flowchart. Please note that the number of included patients equals the number of analyzed patients, as we had no dropouts after inclusion

We screened 84 patients for eligibility, of which 16.7% were excluded (13.1% due to an insufficient bone window; 3.6% due to the wrong diagnosis). The remaining study population consisted of 70 patients (median age: 50.5 years, IQR: 40–58 years; 68.6% female), of which 28.6% (*n* = 20) had a hypoechogenic BR in TCS examination. Two cases needed a consensus decision on raphe echogenicity. Interrater agreement was high (Cohen's kappa = 0.932). Figure 2 depicts the study flowchart.

### Demographics and clinical characteristics

There was no significant difference regarding demographics or clinical features between Long-COVID patients with or without raphe hypoechogenicity. Most patients had a mild course of COVID-19 as assessed by the WHO severity scale (82% with a score of 1 or 2 in the normoechogenic raphe group; 95% in the hypoechogenic raphe group). For detailed information, see Table 1.

**Table 1:** Demographics and clinical characteristics according to raphe status

	Normoechogenic raphe (*n* = 50)	Hypoechogenic raphe (*n* = 20)	*p* value
Age (years)	50 (39–58)	53 (47–58)	0.439^b^
Female sex	68%	70%	0.871^a^
WHO clinical progression scale of COVID-19 according to [13]			0.332^a^
1	4%	0	
2	78%	95%	
3	0	0	
4	10%	5%	
5	8%	0	
Indicators of COVID-19 severity			
Hospital stay	18%	5%	0.262^a^
Intensive care unit	4%	0%	> 0.999^a^
Non-invasive ventilation	0%	0%	> 0.999^a^
Invasive ventilation	0%	0%	> 0.999^a^
Duration between onset and outpatient presentation (days)	190 (110–304)	184.5 (110–299)	0.701^b^
Comorbidities			
Psychiatric or psychosomatic disease	10%	25%	0.135^a^
Hypertension	26%	30%	0.734^a^
Diabetes	6%	5%	> 0.999^a^
Cardiovascular disease	2%	5%	0.493^a^
Cerebrovascular disease	2%	0%	> 0.999^a^
History of cancer	4%	0%	0.586^a^
COPD	4%	0%	0.586^a^
CKD	0%	0%	> 0.999^a^
Thyroid disease	28%	25%	0.799^a^
Neurological disorder	0%	5%^1^	0.286^a^

Continuous variables are given as median and interquartile range and binary variables are given in percent.

^a^Chi-squared test

^b^Mann–Whitney-*U*-Test

^1^One patient with coinciding relapsing–remitting multiple sclerosis

### Symptoms of depression and anxiety

**Fig. 3:**
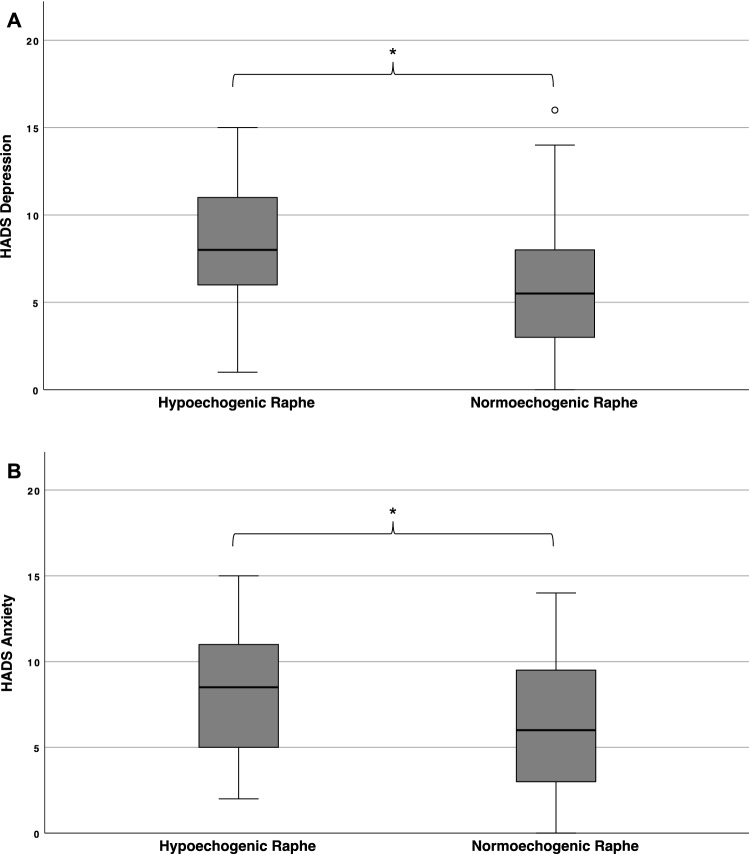
Patients with hypoechogenic raphe scored significantly higher on both HADS subscales of depression (**A**) and anxiety (**B**). Boxplot diagrams with whiskers with a maximum of 1.5 IQR for HADS subscores of depression and anxiety. °Mild outliners (up to 1.5 times of IQR). * Mann–Whitney-*U* test with *p* < 0.05 for the comparison between patients with hypoechogenic versus normoechogenic raphe in TCS

Long-COVID patients with hypoechogenic raphe had significantly higher scores for depression (median 8 versus 5.5, *p* = 0.006) and anxiety (median 9 versus 6.5, *p* = 0.006) compared to patients with normoechogenic raphe (Fig. 3).

Using the predefined cut-offs, 65% of the patients with hypoechogenic raphe showed relevant depressive symptoms as assessed by the HADS at the time of investigation compared to 28% of the patients with a normoechogenic raphe (*p* = 0.004; adjusted OR: 3.884 [1.244–12.123]). The frequency of relevant anxiety symptoms was not significantly different between normoechogenic and hypoechogenic patients (Table 2). Table 3 presents the unadjusted and adjusted ORs for relevant depressive and anxiety symptoms, respectively, applying the predefined cut-offs.

**Table 2:** Symptoms of depression and anxiety

	Normoechogenic raphe (*n* = 50)	Hypoechogenic raphe (*n* = 20)	*p* value
HADS depression	5.5 (3–8)	8 (7–11)	0.006^b^
HADS depression ≥ 8	28%	65%	0.004^a^
HADS anxiety	6.5 (3–10)	9 (5–11)	0.033^b^
HADS anxiety ≥ 8	42%	65%	0.082^a^

Continuous variables are given as median and interquartile range and binary variables are given in percent.

^a^Chi-squared test

^b^Mann–Whitney-*U*-Test

**Table 3:** Odds ratios of relevant depressive and anxiety symptoms

	Unadjusted	Adjusted
HADS depression ≥ 8	4.776 [1.579–14.447]	3.884 [1.244–12.123]
HADS anxiety ≥ 8	2.565 [0.874–7.528]	1.874 [0.583–6.023]

OR [95% CI] for depression and anxiety, respectively, are given unadjusted and adjusted for comorbid psychiatric or psychosomatic disease and COVID-19 severity, using hypoechogenic raphe as the predictor

## Discussion

Until now, it remains unclear whether Long-COVID is a novel, distinct syndrome or whether it is the sum of multiple, overlapping syndromes, with the pathophysiological correlates still being poorly understood [16]. In general, depressive symptoms and emotional disturbances are of high relevance in the post-COVID population. In a report of COVID-19 patients sixty days following discharge, 362 of 1250 patients reported being mildly or moderately emotionally affected by health conditions, and 28 patients reported needing health care related to mental care [17]. In this first study investigating BR alterations in TCS in patients with Long-COVID syndrome, we discovered an independent relationship between hypoechogenic BR and depressive symptoms in this patient population.

This finding raises two hypotheses that could explain the association. Unlikely, TCS alteration of the BR might appear reactive to COVID-19, reflecting changes in the serotonergic systems that remain and cause depressive symptoms as part of Long-COVID syndrome. Interestingly, it has been postulated that modifications of the dopamine and serotonin synthetic pathways might be involved in COVID-19 pathophysiology [10, 11]. In this context, the serotonin reuptake inhibitor fluvoxamine has been proven to reduce clinical deterioration after SARS-CoV-2 infection, indicating a link between serotonin and COVID-19 course [18]. Furthermore, there are similarities in the gene co-expression, co-regulation, and function between Angiotensin I Converting Enzyme 2 (ACE2) and Dopa Decarboxylase (DDC). It has been shown that the mRNA levels of the interferon-inducible truncated isoform of ACE (dACE2) and DDC negatively correlate with the severe acute respiratory syndrome coronavirus type 2 (SARS-CoV-2) virus load in COVID-19 patients [19]. DDC encodes the enzyme that catalyzes the biosynthesis of dopamine, serotonin, and histamine, supporting a possible link between SARS-CoV-2 invasion via ACE2 and the consecutive downregulated expression that might parallel a DDC dysfunction resulting in alterations of the serotonin metabolism [10]. The possibility of a straight DDC dysfunction culminating in a structural correlate is speculative. Nevertheless, it has been suggested that short- and long-term neuropsychiatric disorders, including depression in COVID-19 patients, might partly be explained by a neurotransmitter dysfunction [11]. Therefore, an ongoing dysfunction of the serotonergic neurotransmission after COVID-19 could be responsible for depressive symptoms in Long-COVID patients. There is also evidence for a negative correlation of depression and post-traumatic distress following COVID-19 with grey matter volumes in the anterior cingulate (ACC) and insular cortex as well as axial diffusivity and functional connectivity, supporting the idea that COVID patients might develop alterations in brain structure [20].

On the other hand, a hypoechogenic raphe in TCS might reflect a higher susceptibility to depressive symptoms in the general population. In this context, Walter et al. reported similar prevalence rates of BR alterations in patients with adjustment disorder with depressed mood and patients with major depressive disorder [21]. Individuals with a hypoechogenic raphe might be more vulnerable to developing depressive symptoms due to different events, including critical life events but also acute or chronic diseases such as COVID-19. The inflammatory state that is associated with COVID-19 might be a crucial trigger for continuous depressive symptoms in patients with hypoechogenic raphe. Although the assumption that BR alterations are a risk factor for depressive symptoms cannot be answered conclusively by our cross-sectional study design, it is supported by the similar frequency of a hypoechogenic raphe in our cohort (28.6%) compared to the frequency in healthy controls from various different cross-sectional studies, in which BR hypoechogenicity ranges between 6 and 43% [6, 22]. If the hypothesis that alterations of the brainstem raphe appear reactive to COVID-19 were true, we would expect a higher frequency of this finding in our Long-COVID cohort. Furthermore, we would expect differences in the clinical course of COVID-19 between hypoechogenic and normoechogenic patients, postulating a COVID-19 severity-depending effect of serotonergic dysfunction, which was not seen. Therefore, our data support that a BR hypoechogenicity might be a risk factor for depressive symptoms in Long-COVID. Furthermore, our data do not support a strong association between depressive symptoms in Long-COVID and comorbid psychiatric disease.

There are some limitations to our study. The relatively small sample size might influence the reliability of our findings. However, there were no significant differences in demographics and clinical characteristics between hypoechogenic and normoechogenic patients, suggesting a low risk of confounding. Furthermore, anxiety and depressive symptoms were explored by a self-report measure (HADS). Common limitations of self-report measures, such as introspective limits or the tendency to answer socially desirable need to be taken into account when interpreting these data. General limitations of TCS are the need for a sufficient transtemporal bone window and the reliability of a high-quality ultrasound system, as well as the qualification of the investigator. For our investigation, TCS was conducted by two experienced and DEGUM-certified (German Society for Ultrasound-certified) investigators being blinded to any clinical information of the patients. Therefore, we do not expect a systematic error in this study. Another limitation is associated with the potential of selection bias since we only included Long-COVID patients who presented to our outpatient clinic. Overall, these patients experienced a mild COVID-19 disease course. More severely affected patients might have been missed because of the inability to present to our outpatient clinic due to care-dependency or motivational problems. This should also be considered when interpreting the data.

## Conclusions

In conclusion, the frequency of a hypoechogenic brainstem raphe in Long-COVID patients does not differ from the healthy population but is independently associated with depressive symptoms in these patients. A longitudinal study design is needed to answer whether raphe alterations are direct consequences of COVID-19 or if they a priori reflect a higher susceptibility for depressive symptoms in case of specific events; the latter is seen as more probable by the authors. Nevertheless, our data indicate that TCS could be a promising tool for predicting depressive symptoms in patients with Long-COVID syndrome, facilitating to include psychiatric support and therefore streamlining therapeutic algorithms. Furthermore, it might help to improve the categorization and understanding of the spectrum and pathophysiology of Long-COVID syndrome.

## Supplementary Information

Below is the link to the electronic supplementary material.

Supplementary file1 (DOCX 17 KB)

Supplementary file2 (DOCX 32 KB)

## Data Availability

The data that support the findings of this study are available from the corresponding author upon reasonable request.
