# Supplementary material for: Hypoechogenicity of brainstem raphe in long-COVID syndrome–less common but independently associated with depressive symptoms: a cross-sectional study
Source: J Neurol. 2022 May 12;269(9):4604–10. doi: 10.1007/s00415-022-11154-3 (PMC9098142; doi:10.1007/s00415-022-11154-3)
Supplement: Supplementary file 1 — Supplementary file1 (DOCX 17 KB) [file 415_2022_11154_MOESM1_ESM.docx]

**Table S1: WHO clinical progression scale (WHO Working Group, 2020)**

| **Patient State** | **Descriptor** | **Score** |
| --- | --- | --- |
| **Uninfected** | Uninfected; no viral RNA detected | **0** |
| **Ambulatory mild disease** | Asymptomatic; viral RNA detected | **1** |
|  | Symptomatic; independent | **2** |
|  | Symptomatic; assistance needed | **3** |
| **Hospitalised: moderate disease** | Hospitalized; no oxygen therapy* | **4** |
|  | Hospitalized; oxygen by mask or nasal prongs | **5** |
| **Hospitalised: severe disease** | Hospitalized; oxygen by NIV or high flow | **6** |
|  | Intubation and mechanical ventilation, pO_2_/FiO_2_ ≥150 or SpO_2_/FiO_2_ ≥200 | **7** |
|  | Mechanical ventilation pO_2_/FiO_2_ <150 (SpO_2_/FiO_2_ <200) or vasopressors | **8** |
|  | Mechanical ventilation pO_2_/FiO_2_ <150 and vasopressors, dialysis or ECMO | **9** |
| **Dead** | Dead | **10** |

ECMO=extracorporeal membrane oxygenation. FiO_2_=fraction of inspired oxygen. NIV=non-invasive ventilation. pO_2_=partial pressure of oxygen. SpO_2_=oxygen saturation. *If hospitalised for isolation only, record status as for ambulatory patient.
